# Amycolatopsis ponsaeliensis sp. nov., a novel endophytic actinobacterium isolated from the root nodules of Alnus glutinosa

**DOI:** 10.1099/ijsem.0.006810

**Published:** 2025-06-13

**Authors:** Ryan Michael Thompson, Edward M. Fox, Georgios Koutsidis, Maria del Carmen Montero-Calasanz

**Affiliations:** 1 School of Natural and Environmental Sciences, Newcastle University, Newcastle upon Tyne, NE1 7RU, UK; 2 Department of Applied Sciences, Northumbria University, Newcastle upon Tyne, NE1 8ST, UK; 3 IFAPA Las Torres-Andalusian Institute of Agricultural and Fisheries Research and Training, Junta de Andalucía, Cra. Sevilla-Cazalla, km 12.2. 41200, Alcalá del Río, Seville, Spain

**Keywords:** *Alnus glutinosa*, *Amycolatopsis*, endophyte, novel species, phytoremediation, plant growth promotion

## Abstract

The root nodules of *Alnus glutinosa* remain a relatively understudied niche, with poorly described associated microbial communities. In this study, the isolate RTGN1^T^ was recovered from root nodules collected from Gateshead, UK, and was identified as belonging to *Amycolatopsis* based on 16S rRNA gene similarity and phylogenomic placement. This isolate was polyphasically characterized, displaying the ability to grow between 12 and 28 °C and pH 6 and 8 and exhibiting the genes necessary to produce the polar lipids phosphatidylethanolamine and phosphatidylglycerophosphate, alongside DL-type peptidoglycan, which are diagnostic of *Amycolatopsis*. Overall genomic relatedness index values were below the cut-off value for delineating a novel species. As such, it is proposed that RTGN1^T^ be recognized as the type strain (=CECT 30870^T^=CABI 507287^T^) of *Amycolatopsis ponsaeliensis* sp. nov. The RTGN1^T^ isolate was screened using *in silico* and *in vitro* methods and was found to possess a number of genes and pathways related to secondary metabolite production and plant growth promotion. Such genes may serve as an avenue of future study regarding biotechnological potential and use as a bioinoculant to increase phytoremediation efficiency.

## Introduction

*Alnus glutinosa* is a tree species within the actinorhizal plant group, with this group consisting of 24 genera across 8 families of angiosperms [1]. These plants are distributed across a wide range of environments. The ability to inhabit a wide range of habitats is in part due to the symbiotic association with the nodule-forming *Frankia*ceae [1,2]. *Frankia*ceae are renowned for their ability to fix nitrogen, with estimations proposing that the *Frankia*ceae-actinorhizal symbiosis can fix 240–350 kg ha^−1^ of nitrogen per year [3,4]. The nitrogen fixation of this symbiosis often renders *Alnus* a pioneer species upon marginalized land. *Alnus* is of interest for its application to bioremediate such sites, as *Alnus* exhibits the ability to improve soil nutrients while removing pollutants from the soil [5].

However, despite the interest in these trees, the root nodule microbiome remains understudied, with the bulk of the research to date focused upon *Frankia*ceae. Aside from *Frankiaceae*, a range of other micro-organisms are capable of bioremediation processes. Thus, isolation was attempted from the root nodules of these trees to study the non-*Frankiaceae* inhabitants of the nodules and the possible roles they may play in the roots of these plants [6].

The *Amycolatopsis* genus, within the family of *Pseudonocardiaceae*, was first proposed by Lechevalier *et al*. [7], with subsequent emendations by Lee [8], Tang *et al*. [9] and most recently Nouioui *et al*. [10]. At the time of writing, the *Amycolatopsis* genus is composed of 94 valid species [11], with a number of these noted as plant endophytes such as *Amycolatopsis suaedae* and *Amycolatopsis samaneae* [12,13]. *Amycolatopsis* are noted as gifted producers of secondary metabolites; examples of such include vancomycin and rifamycin [14].

As *Amycolatopsis* is a well-characterized genus in terms of cellular composition with the main chemotaxonomy constituents being well documented [15], *in vitro* chemotaxonomy may be considered redundant, due to the lower throughput, higher expense and having somewhat unreliable reproducibility [16]. In such cases, *in silico* chemotaxonomy has emerged as an alternative and powerful tool for the classification of well-characterized taxonomic groups, with this method being successfully applied to the characterization of species such as *Ningiella ruwaisensis* [17] and *Parapseudiflavitalea muciniphila* [18], alongside the characterization of novel genera in the family *Geodermatophilaceae* [19].

In this study, a novel endophytic *Amycolatopsis* isolated from the root nodules of an *A. glutinosa* was characterized using a polyphasic approach integrating genome-scale data. The location of novel species in this genus is of interest to derive novel secondary metabolites useful for pharmaceutical and industrial application. In addition, the isolate was screened for plant growth promotion (PGP) attributes such as phosphate solubilization, siderophore production and antibiotic production.

## Methods

### Bacterial isolation, cultivation, maintenance and morphological and physiological analysis

Strain RTGN1^T^ was isolated from *A. glutinosa* following the methods detailed in Thompson *et al*. [20], from nodules collected from a tree located close to the boathouse building within Saltwell Park (54° 56′ 41.003″ N 1° 36′ 21.067″ W). After achieving an axenic culture, RTGN1^T^ was maintained upon GYM *Streptomyces* agar plates (DSMZ medium no. 65) with long-term storage at −80 °C in 30% v/v glycerol. For morphological characteristics, RTGN1^T^ was grown axenically upon GYM *Streptomyces* agar and incubated at 28 °C for 7 days.

The ability of RTGN1^T^ to grow at various temperatures was assayed by growing RTGN1^T^ upon GYM *Streptomyces* agar at 4, 12–14, 28, 37 or 45 °C for 2 weeks, after which growth was recorded based on visual inspection. The abilities of RTGN1^T^ to grow at various pH conditions were tested by growing RTGN1^T^ upon GYM *Streptomyces* agar between the pH ranges of 4–10 at 28 °C for 2 weeks, with the respective buffering agents detailed in Xu *et al*. [21] added to the medium, and the CaCO_3_ of the medium was removed. In the case of the pH 4 and 5 tests, these were conducted using 50 ml of GYM *Streptomyces* broth, due to the low pH, making the solid agar susceptible to tearing during inoculation.

The ability of the isolate to utilize various carbon and nitrogen sources and withstand differing concentrations of NaCl and its susceptibility to antibiotics were determined using the OmniLog instrument (Biolog Inc.) and GEN-III plates. Isolate biomass was prepared by growing RTGN1^T^ upon GYM *Streptomyces* agar plates at 28 °C for 5 days. Loops of biomass from the agar plate were transferred to an Eppendorf tube containing 1 ml of IF-C solution (Biolog Inc.). The IF-C solution tubes were then adjusted to 90% transmittance by adding drops of the homogenous suspension. The GEN-III plates were inoculated following the manufacturer’s instructions, sealed with parafilm to prevent evaporation and incubated within an OmniLog at 28 °C for 7 days. Two technical replicates were used for each isolate, with the results analysed using the opm package (version 1.2.33) within the R statistical software environment [22]. Reactions that reflected different behaviour between replicates were regarded as ambiguous.

### 16S rRNA gene identification and phylogenetic analysis

DNA was extracted from RTGN1^T^ for preliminary identification. Briefly, a loop of axenic culture material was collected, placed in 200 µl of sterile distilled water and vortexed (Vortex Genie2, Scientific Industries) at maximum speed for 15 min. After which, the material was heated at 95 °C for 15 min (Compact digital dry bath/block heater, Thermo Scientific™) and then placed on ice for 10 min. The lysate was briefly centrifuged at 10,000 ***g*** (micro star 17R, VWR), and 2 µl of supernatant served as the template for PCR.

The 16S rRNA gene was amplified using the 27F (5′-AGAGTTTGATCMTGGCTCAG-3′) and 1492R (5′-TACGGYTACCTTGTTAYGACTT-3′) primers [23]. The PCR was composed of 2 µl of template, 1 µl of 10 µM 27F primer, 1 µl of 10 µM 1492R primer, 12.5 µl of 2X MyFi™ master mix (Bioline) and 8.5 µl of Mili-Q water. The settings of the PCR cycles were as follows: initial denaturation at 95 °C for 60 s, followed by 35 cycles of 95 °C for 30 s, 57.3 °C for 20 s and 72 °C for 45 s and a final elongation step at 72 °C for 120 s (TC-412 thermal cycler, Techne).

The PCR product was analysed using gel electrophoresis with a 1% agarose-1X TBE gel containing GelRed® (5 µl/100 ml), with DNA separated at 90 V for 90 min (PowerPack 300, Bio-Rad). After electrophoresis, the gel was visualized using a Gel Doc™ EZ gel documentation system (Bio-Rad), with the amplified DNA compared to the GeneRuler DNA ladder mix, ready-to-use ladder (Thermo Scientific).

The PCR product was then purified using ExoSAP-IT™ (Applied Biosystems) and prepared for sequencing according to Eurofins Ltd. LightRun tube service. The returned 16S rRNA gene sequences were checked for complementarity and where appropriate reverse complemented (http://www.reverse-complement.com/). Forward and reverse sequences were aligned using the Multiple Sequence Comparison by Log-Expectation (muscle) tool on the European Molecular Biology Laboratory–European Bioinformatics Institute (EMBL-EBI) server (https://www.ebi.ac.uk/Tools/msa/muscle/) with all settings selected by default [24,25].

The consensus sequence was uploaded to the 16S-based ID tool of the EzBioCloud server (https://www.ezbiocloud.net/) [26] to determine the isolates’ 25 nearest validly named neighbours. The neighbours were utilized to infer the isolate phylogeny, with the type strain of *Thermotunica guangxiensis* serving as an outgroup. The 16S rRNA gene phylogenetic tree was constructed using the Genome-to-Genome Distance Calculator (GGDC) server using the Leibniz-Institut Deutsche Sammlung von Mikroorganismen und Zellkulturen GmbH (DSMZ) phylogenomic pipeline adapted for single genes (https://ggdc.dsmz.de/phylogeny-service.php) [27,29]. The sequences were aligned using muscle, with maximum likelihood and maximum parsimony trees inferred from this alignment using Randomized Axelerated Maximum Likelihood (RAxML) and Tree Analysis Technology (TNT), respectively [30,32]. For maximum likelihood, rapid bootstrapping in conjunction with the autoMRE bootstopping criterion and subsequent search for the best tree was used [33]. For maximum parsimony, 1,000 bootstrapping replicates were used in conjunction with tree-bisection-and-reconnection branch swapping and 10 random sequence addition replicates, and the sequences were checked for a computational bias using the X^2^ test as implemented in Phylogenetic Analysis Using Parsimony (PAUP*) [34].

### DNA extraction, *de novo* genome assembly and phylogenomic analysis

DNA was extracted, sequenced and assembled as detailed in Thompson *et al*. [20]. The resulting genome assembly was uploaded to the Type Strain Genome Server (TYGS) (https://tygs.dsmz.de) to determine the ten nearest type species to RTGN1^T^ based on 16S rRNA gene and genome relatedness [27,30, 35,41]. To supplement the digital DNA–DNA hybridization (dDDH) obtained from TYGS, the average nucleotide identity (ANI) was calculated using the ANI calculator available on the EzBioCloud webserver, which utilizes the OrthoANIu algorithm (https://www.ezbiocloud.net/tools/ani) [42,43]. The ANI values were generated by comparing the genome of RTGN1^T^ to the list of near neighbours generated by TYGS.

### *In silico* chemotaxonomy

The genome assembly of RTGN1^T^ was uploaded to the Integrated Microbial Genomes Genomes Online Database (IMG GOLD) and annotated using the IMG annotation pipeline version 5.1.8 [44,46]. *In silico* chemotaxonomy was conducted primarily using the IMG genome annotation, specifically the Kyoto Encyclopedia of Genes and Genomes (KEGG) database [47]. Genes which were not annotated within the KEGG pathways were searched for within the protein family (Pfam) database annotated by IMG or located using the Basic Local Alignment Search Tool (blast) function within IMG. In order to do this, query sequences from the isolates’ near neighbours, or the wider genus/family, were located by searching the gene name/enzyme class in the Universal Protein (UniProt) database [48,49].

### Genome analysis

Mobile genetic elements were located within the assembly using several tools. The first of which was Phage Search Tool Enhanced Release (PHASTER) [50,51], using the bacteria, prophage/virus, and DNA fragment databases (version release date 22 December 2020, 22 December 2020, and 29 March 2018, respectively). The PlasmidFinder 2.1 tool [52,53] was used to identify plasmids within the assembly (software version 2.0.1, database version 2021-11-29). Within this tool, both the Gram-positive and *Enterobacteriales* databases were utilized, with a threshold for minimum percentage identity set to 95% and the maximum coverage set to 60%. CRISPRFinder (version 1.1.2) [54,56] was used to locate clustered regularly interspaced short palindromic repeats (CRISPR) sequences, with settings left as standard. Biosynthetic gene clusters were identified using the antiSMASH tool (version 6.1.1) [57], with this tool run in the ‘relaxed’ setting with all the extra features enabled.

Antimicrobial resistance genes were searched within the RTGN1^T^ genome using ResFinder 4.1 and Resistance Gene Identifier (RGI) (version 5.2.1) [58,60]. The ResFinder 4.1 tool was used to locate all available resistance genes, which were possible to be searched for within this tool, with the threshold for identification set to 90% and the minimum length set to 60%. The RGI tool was run upon the Comprehensive Antibiotic Resistance Database (CARD) (version 3.2.4) [59], with perfect and strict hits only, and the use of the ‘nudge’ ability was excluded.

In addition to the IMG annotation, the assembly of RTGN1^T^ was uploaded to the Rapid Annotation using Subsystem Technology (RAST) (version 2.0) server and annotated using the RASTtk pipeline using the genetic code 11 for bacteria. The RASTtk annotation process was enabled to automatically fix errors, and all other settings were left as default [61,63]. The RAST annotation was used to locate genes related to the modulation of phytohormones, with the query gene sequences being located on UniProt [48,64] and the NCBI [65].

### PGP activity assays

The ability to degrade 1-aminocyclopropane-1-carboxylic acid (ACC) was assayed using the media detailed in Patil *et al*. [66]. The media utilized consisted of 0.4 g l^−1^ CaCO_3_, 2 g l^−1^ glucose, 2 g l^−1^ sodium citrate, 2 g l^−1^ potassium gluconate, 15 g l^−1^ agar and 3 mM ACC (filtered through a 0.2 µm filter, added after autoclaving). The amount of CaCO_3_ utilized in the current study was reduced from 4 to 0.4 g l^−1^, to maintain a stable pH of 6. The media had a dark yellow and pale blue–green colour in the case of phenol red and bromothymol blue, respectively, with a change in pH yielding a colour change to red and blue, respectively. Prior to testing RTGN1^T^ upon the ACC degradation assay media, the same medium was created with 3 mM of ACC replaced with 2 g l^−1^ of (NH_4_)_2_SO_4_; this is a readily metabolized nitrogen source and would confirm if RTGN1^T^ could grow upon this medium [67]. RTGN1^T^ was streaked upon this (NH_4_)_2_SO_4_-containing medium and incubated for 2 weeks at 28 °C. After which, RTGN1^T^ was streaked upon the ACC-containing medium and grown for 2 weeks at 28 °C to observe growth and change in media pH.

The ability of RTGN1^T^ to solubilize phosphate and produce siderophores was assayed using the National Botanical Research Institute’s phosphate growth (NBRIP) medium and chrome azurol S (CAS) media, respectively [68,69]. For both the NBRIP and CAS media, an agar plug from a growing culture of RTGN1^T^ was placed upon each media type, with the colony material facing down. The inoculated CAS and NBRIP were incubated for 2 weeks at 28 °C, with any zones of activity, recorded by measuring the radius from the edge of the plug to the edge of the activity zone, alongside the entire diameter of the activity zone.

The ability to produce indole-3-acetic acid (IAA) from tryptophan was analysed using the methods of Patten and Glick [70]. In brief, RTGN1^T^ was cultivated in nutrient broth supplemented with 100 mg l^−1^ tryptophan, for 7 days at 28 °C, shaking at 180 r.p.m. After cultivation, the culture was centrifuged at 2,057 ***g*** for 5 min (Centrifuge 5810 R, Eppendorf), and 0.5 ml of the supernatant was removed and added to 2 ml of Salkowski’s reagent (150 ml concentrated H_2_SO_4_, distilled H_2_O and 7.5 ml 0.5 M FeCl_3_). A blank consisting of 0.5 ml of nutrient broth containing 100 mg l^−1^ tryptophan was also prepared with 2 ml of Salkowski’s reagent added. A standard curve from 0 to 200 p.p.m. of IAA was created using IAA powder (Sigma-Aldrich) with 2 ml of Salkowski’s reagent added. The blank, sample and standards were incubated for 20 min, after which the absorbance was read at 535 nm (Spectrophotometer model 4101, Zuzi). The standard data points were plotted graphically, and a linear trend line was fitted. The absorbance of the inoculated samples was compared to this trendline, giving a rough approximation regarding the amount of IAA produced by the isolates.

### Analysis of metabolites

Compounds produced by RTGN1^T^ were analysed using liquid chromatography MS (LC-MS). This was conducted by growing RTGN1^T^ in 50 ml of liquid medium at 28 °C shaking at 150 r.p.m. (KS 4000 i control, IAK) for 4 days until the stationary phase was reached. The media supernatant was obtained by centrifuging the culture at 10,000 ***g*** for 5 min (Sorvall Legend X1R Centrifuge, Thermo Scientific). In addition, an uninoculated culture of GYM *Streptomyces* broth was incubated for 13 days, as a control. From these media supernatants, 5 ml was freeze dried and reconstituted in 5 ml of methanol, followed by filtration through a 0.2 µm syringe filter and transferred to 1 ml chromatography vials.

The LC-MS analysis was performed using a Thermo Scientific Vanquish Liquid Chromatography front end connected to IDX High Resolution Mass Spectrometer system. The MS data was acquired using the AcquieX acquisition workflow. The operational parameters of the MS orbitrap detector were as follows: MS1 mass resolution 60K, MS2 mass resolution 30K, collisional stepped energy set at 10 (low), 30 (mid), and 50 (high), mass scan range at 100–1000 m/z and radio frequency lens at 35%. The automatic gain control target mode was set at custom and the normalised automatic gain control target was set to 25% (100%=3^6^) with a maximum injection time of 50 ms. The intensity threshold was set to 2^4^, with all data acquired in profile mode. A corresponding extraction blank was used to create a background exclusion list, and a pooled quality control was used to create the inclusion list.

The chromatographic separation was conducted using a Waters Acquity UPLC BEH amide column (2.1×150 mm, with a particle size of 1.7 µm) and operated at 45 °C with a flow rate of 250 µl min^−1^. The liquid chromatography gradient consisted of a binary buffer system composed of buffer A (95%/5% LC-MS grade water/acetonitrile) and buffer B (95%/5% acetonitrile/LC-MS grade water), with both buffers containing 10 mM ammonium formate.

Independent buffer systems were used for both the positive and negative modes. For the positive mode, the pH of the buffers was adjusted using 0.1% formic acid, while for the negative mode, the pH was adjusted using 0.1% ammonia solution. The liquid chromatography gradient was the same for both polarities; 95% of buffer B at T0, held for 1.5 min, linearly decreased to 50% at 11 min, held for 4.5 min, returned to the starting condition, and held for a further 4.5 min (column stabilization). Injection volume was maintained at 3 µl for the positive mode and 5 µl for the negative mode, with 3.5 and 2.5 kV being applied in the positive and negative modes, respectively. The heated electrospray ionization conditions for 250 µl min^−1^ were as follows: sheath gas: 35, aux gas 7, sweep gas 0, ion transfer tube temperature 300 °C and vaporizer temperature 275 °C.

The hydrophilic interaction liquid chromatography-positive and -negative data sets were processed using Compound Discoverer 3.2 (database to online m/z cloud database: mass tolerance 10 p.p.m., maximum shift 0.3 min, alignment model set to adaptive curve, minimum intensity 500 K, S/N threshold of 3, compound consolidation and retention time tolerance 0.3 min). Database matching was performed at MS2 level with a similar index of 70% or better.

Regarding quality control, hydrophilic interaction liquid chromatography pooled quality control samples were used to assess instrument drift. One quality control was conducted for every ten injections. A quality control reproducibility assessment cut-off limit of 15% or less was deemed acceptable. Metabolite features with a relative sd of 25% or less across the quality control during the data-dependent acquisition analysis were retained during Peak table quality assessment followed by MS2 matching and chemical formula determination. The resulting data was analysed by calculating the amount of the compound present in the microbial sample relative to the media control, with compounds of interest above 50% presence in the microbial sample relative to the control considered for discussion.

## Results and discussion

### Phylogenetic analysis

**Fig. 1.**
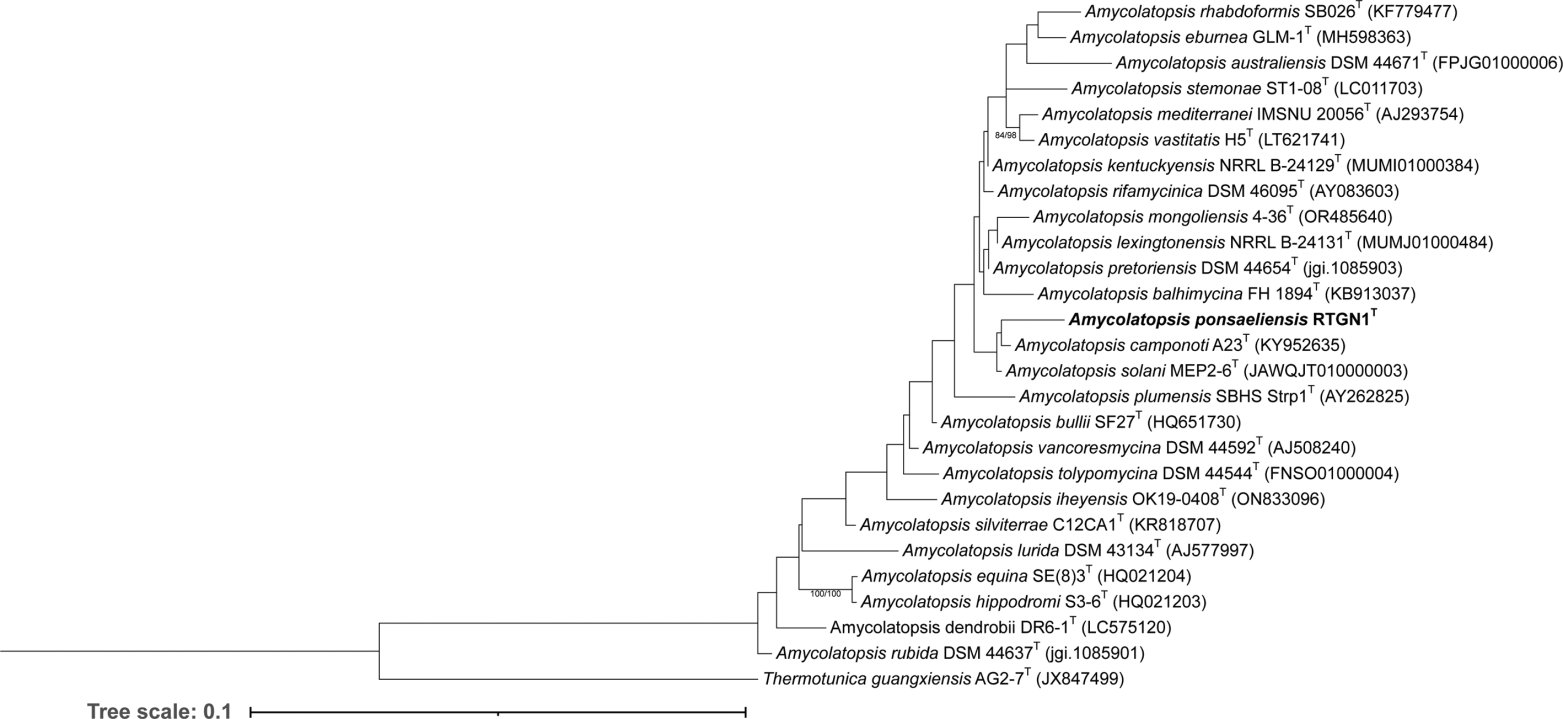
16S rRNA gene phylogenetic tree of *Amycolatopsis* sp. RTGN1^T^ and related *Amycolatopsis* species. Maximum likelihood tree inferred under the GTR+GAMMA model and rooted using midpoint rooting. The branches are scaled in terms of the expected number of substitutions per site. The numbers above the branches are support values for maximum likelihood (left) and maximum parsimony (right) bootstrapping, with only values greater than 60% being displayed. Image processing was conducted using the interactive tree of life tool [116].

Based on the 16S rRNA gene phylogenetic analysis, RTGN1^T^ did not cluster alongside its near neighbour *Amycolatopsis rhabdoformis* (98.96% 16S rRNA gene similarity). It instead clustered next to *Amycolatopsis camponoti* (98.94% 16S rRNA gene similarity) (Fig. 1 and Table S1, available in the online Supplementary Material). However, it is important to note that the majority of the bootstrap values were below 60%, which does not provide robust support for the observed topology. Nevertheless, despite the poor branch support, RTGN1^T^ was unambiguously placed within the *Amycolatopsis* clade.

Sequencing of the RTGN1^T^ genome yielded 10,687,744 reads with 10,660,346 reads left after filtering. The resulting assembly had a G+C content of 70.9 mol% and was 11,990,019 bp in length, composed of 147 contigs, with these contigs having an N_50_ value of 179,211 and an estimated sequencing depth of 134X.

**Fig. 2.**
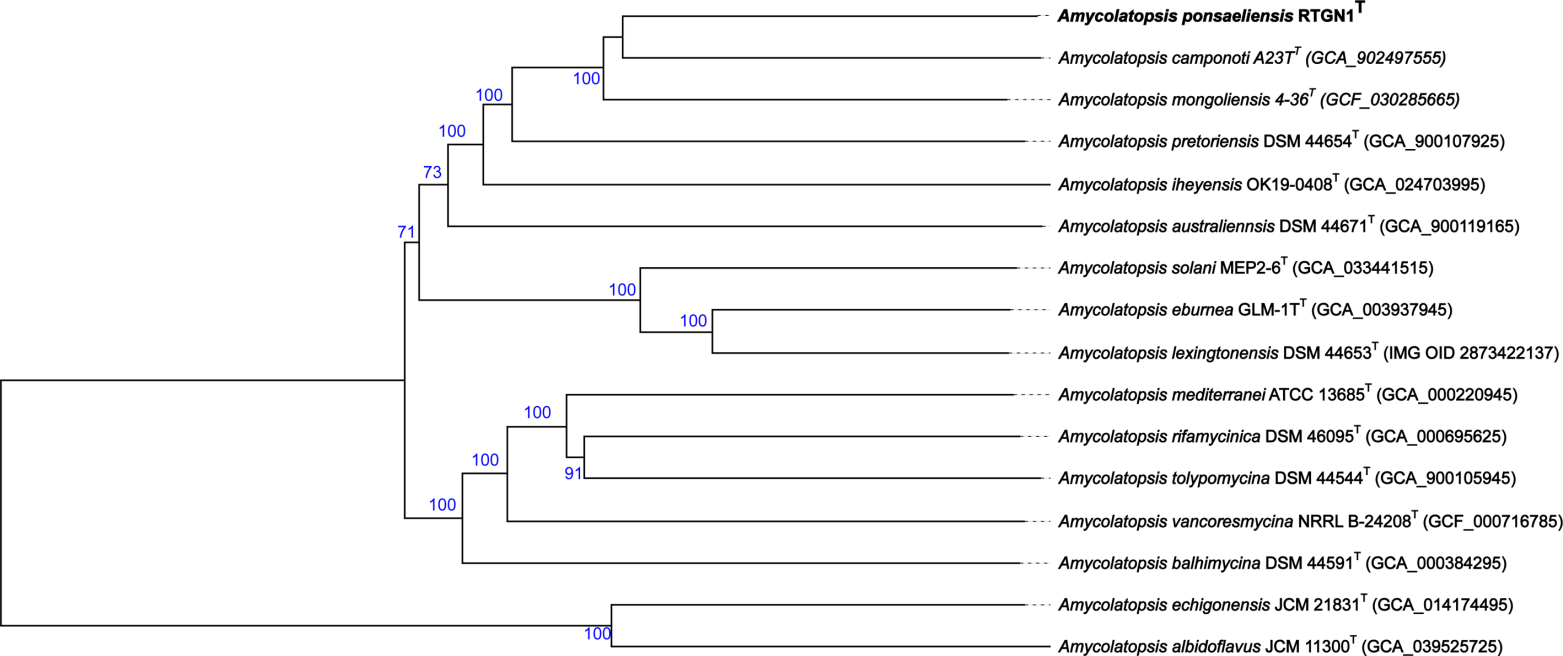
Genome-based phylogenetic tree of *Amycolatopsis* sp. RTGN1^T^ and related *Amycolatopsis* species created using the TYGS database. Tree inferred with FastME 2.1.6.1 [39] from GBDP distances calculated from genome sequences. The branch lengths are scaled in terms of GBDP distance formula d_5_. The blue numbers above the branches are GBDP pseudo-bootstrap support values >60% from 100 replications, with an average branch support of 91.2%. The tree was rooted at the midpoint [36].

The phylogenomic analysis of RTGN1^T^ displayed greater bootstrap support than the 16S rRNA gene-based phylogeny (Fig. 2). Unlike the 16S rRNA gene phylogeny, the phylogenomic analysis placed *Amycolatopsis* sp. RTGN1^T^ adjacent to *A. camponoti* and in a clade also alongside *Amycolatopsis mongoliensis* 4-36^T^. In terms of similarity based on the dDDH and ANI (Table 1), it appeared that RTGN1^T^ was more closely related to *A. mongoliensis* (46.2% and 91.90 %, respectively) than *A. camponoti* (45.2% and 91.66%, respectively). However, the placement of RTGN1^T^ next to *A. camponoti* may be due to the low bootstrap support of this branch. Nevertheless, both dDDH and ANI similarity values demonstrated that RTGN1^T^ is below those used to demarcate a novel species (<70% for dDDH as calculated in [28] and 95–96% for ANI as indicated by [71]).

**Table 1.** The dDDH and ANI percentage similarity results of *Amycolatopsis* sp. RTGN1^T^, in comparison to near neighbours

	dDDH	ANI
*A. mongoliensis* 4-36^T^	46.2	91.90
*A. camponoti* A23^T^	45.2	91.66
*Amycolatopsis pretoriensis* DSM 44654^T^	39.0	89.69
*Amycolatopsis iheyensis* OK19-0408^T^	36.9	88.91
*Amycolatopsis australiensis* DSM 44671^T^	35.6	88.40
*Amycolatopsis lexingtonensis* DSM 44653^T^	34.8	88.18
*Amycolatopsis solani* MEP2-6^T^	34.8	88.35
*Amycolatopsis eburnea* GLM-1T^T^	34.5	88.11
*Amycolatopsis balhimycina* DSM 44591^T^	34.2	87.63
*Amycolatopsis mediterranei* ATCC 13685^T^	34.1	87.54
*Amycolatopsis rifamycinica* DSM 46095^T^	33.6	87.4
*Amycolatopsis vancoresmycina* NRRL B-24208^T^	33.5	87.48
*Amycolatopsis tolypomycina* DSM 44544^T^	33.2	87.27
*Amycolatopsis echigonensis* JCM 21831^T^	22.7	79.28
*Amycolatopsis albidoflava* JCM 11300^T^	22.4	77.93

### Phenotypic testing

Upon GYM *Streptomyces* agar, RTGN1^T^ exhibited a dark yellow/brown substrate mycelium with white, powdery aerial mycelium. The colonies are 1–4 mm in size, with a round to somewhat irregular form, flat elevation and white hyphal margins. This description is in accord with its five nearest neighbours *A. mongoliensis* 4-36^T^ [72], *A. camponoti* A23^T^ [73], *Amycolatopsis pretoriensis* NRRL B-24133^T^ [74], *Amycolatopsis iheyensis* OK19-0408^T^ [75] and *Amycolatopsis australiensis* GY048^T^ [76].

RTGN1^T^ displayed the ability to grow between the temperature range of 12–28 °C and within the pH range of 6–8, with optimal growth observed at 28 °C and pH 6–7. This was within the ranges observed for the nearest neighbour *A. mongoliensis* 4-36^T^, which exhibited growth between 15 and 37 °C and pH range of 4.5–12 [72]. Likewise, *A. camponoti* A23^T^ displayed optimum growth between 28 and 30 °C and pH 6–9 [73], which is comparable to RTGN1^T^.

According to Biolog GEN III Microplates, RTGN1^T^ was capable of utilizing glucose, fructose and galactose, which are traits shared by its three nearest neighbours *A. mongoliensis* 4-36^T^, *A. camponoti* A23^T^ and *A. pretoriensis* NRRL B-24133^T^ (Table 2). However, RTGN1^T^ was found to possess a number of differences regarding carbon and nitrogen source utilization compared to these neighbours. RTGN1^T^ was unable to utilize l-arginine, l-alanine, l-histidine, mannitol and melibiose, all of which are utilized by its nearest neighbour *A. mongoliensis* 4-36^T^. Likewise, RTGN1^T^ differed from *A. camponoti* A23^T^, as RTGN1^T^ exhibited the ability to utilize d-salicin, which *A. camponoti* A23^T^ did not, and *A. camponoti* A23^T^ was capable of degrading gelatin, which RTGN1^T^ was unable to. RTGN1^T^ also differed from *A. pretoriensis* NRRL B-24133^T^, as RTGN1^T^ did not exhibit the ability to utilize melibiose and gelatin, which *A. pretoriensis* NRRL B-24133^T^ did. Such observed similarities and differences should nevertheless be taken with caution, as different methods to those employed in the current study were used and therefore, under standardized conditions, results may vary.

**Table 2.** Carbon and nitrogen source utilization characteristics of *Amycolatopsis* sp. RTGN1^T^ and related *Amycolatopsis* species. Data regarding the metabolic abilities of *A. mongoliensis* 4-36^T^, *A. camponoti* A23^T^ and *A. pretoriensis* NRRL B-24133^T^ was gathered from Zakalyukina *et al*. [73], Labeda *et al*. [74] and Ngamcharungchit *et al*. [75], respectively. +, Positive activity; −, negative activity; n/a, data not available within the literature

Characteristic	*Amycolatopsis* sp. RTGN1^T^	*A. mongoliensis* 4-36**^T^**	*A. camponoti* A23^T^	*A. pretoriensis*NRRL B-24133^T^
l-Arginine	−	+	n/a	n/a
l-Alanine	−	+	n/a	n/a
l-Histidine	−	+	n/a	n/a
Mannitol	−	+	−	−
d-Mannitol	−	+	+	−
Melibiose	−	+	n/a	+
d-Salicin	+	+	−	+
Gelatin	−	n/a	+	+
d-Glucose	+	+	+	+
d-Fructose	+	+	+	+
d-Galactose	+	+	+	+

### *In silico* chemotaxonomy

Regarding menaquinones, *Amycolatopsis* representatives are generally noted as containing di-, tetra- or hexahydrogenated menaquinones with nine isoprene units [15]. As annotated by IMG, RTGN1^T^ contained a menaquinone-producing pathway composed of *menABCDEFGH* [77]. However, genes encoding vitamin K-dependent gamma-carboxylase (EC: 4.1.1.90) and vitamin K epoxide reductase (warfarin-sensitive) (EC: 1.17.4.4) were absent, which seems a common feature of the genus based on searches of these genes in other species on UniProt. It is noted that *menJ*, which RTGN1^T^ contains, could conduct the function of vitamin K-dependent gamma-carboxylase (EC: 4.1.1.90) and vitamin K epoxide reductase (warfarin-sensitive) (EC: 1.17.4.4) [78], thus allowing the production of menaquinones. The fatty acid profile of *Amycolatopsis* is noted as primarily consisting of 14-methyl-pentadecanoic acid alongside hexadecanoic, 12-methyltridecanoic, 13-methyltetradecanoic, heptadecanoic and octadecanoic acids [15]. RTGN1^T^ was annotated as containing the majority of the components of the type II fatty acid synthase, including *fabDHFGZF* and an acetyl-CoA carboxylase biotin carboxylase carrier protein (EC: 6.4.1.2). In addition, it carried the *fabI* and fatty acyl-acyl carrier protein thioesterase B encoding genes (Table S2).

Pertaining to polar lipids, the major polar lipids of *Amycolatopsis* are phosphatidylethanolamine and phosphatidylglycerol, with variable occurrence of diphosphatidylglycerol, hydroxyphosphatidylethanolamine, phosphatidylinositol, phosphatidylinositol mannosides, phosphatidylserine and phosphatidylmethylethanolamine [15]. RTGN1^T^ seemed able to produce one of the major phospholipids, phosphatidylethanolamine as annotated by the IMG KEGG pathway. However, there was no gene present in the annotated pathway to allow the conversion of sn-glycerol-3-phosphate to 1-acyl-sn-glycerol-3-phosphate. A 1-acyl-sn-glycerol-3-phosphate acyltransferase encoding gene, to conduct this function, was located using blast (Table S2). In addition, a gene encoding phosphatidylglycerophosphatase B (EC: 3.1.3.27) was located using blast, with this gene allowing the production of phosphatidylglycerol from phosphatidylglycerophosphate, with phosphatidylglycerol being the other major polar lipid of *Amycolatopsis* [15].

In the case of peptidoglycan, RTGN1^T^ contains *murABCDEFYG* required for the production of dl-type peptidoglycan, which is diagnostic of *Amycolatopsis* [15]. RTGN1^T^ also contained the necessary dd-transpeptidase (dd-TPase) and dd-carboxypeptidase (dd-CPase) to allow cross-linkage of neighbouring peptidoglycan strands [79]. However, the glycosyltransferase (GTase) used to synthesize the peptidoglycan strand was not annotated, and this was located using blast, which returned a match with 87% identity. However, as RTGN1^T^ also contains a *dapF*, a diaminopimelate epimerase (EC: 5.1.1.7), the production of either dl or ll-diaminopimelic acid may be possible, despite *Amycolatopsis* usually only containing dl-diaminopimelic acid [15,80].

### Generalized genome characterization

The assembly of RTGN1^T^ was annotated as containing 11,499 genes with 11,345 of these being protein coding genes with 8,221 having an identified function. No plasmids were identified in RTGN1^T^. However, eight confirmed CRISPR genes were located, with 17 possible CRISPR sequences also located (Table S3). In addition, two putative incomplete prophages were identified, named PHAGE_Bacill_G_NC_023719(2) and PHAGE_Thermu_IN93_NC_004462(1), consisting of 10 and 11 proteins, respectively.

### Antimicrobial resistance

Based on the analysis using RGI with the CARD database, RTGN1^T^ seemed to possess the glycopeptide resistance genes *vanHOX*. These genes function to replace d-alanyl-d-alanine used in the synthesis of peptidoglycan with d-alanyl-d-lactate, which is resistant to the action of glycopeptide antibiotics [81,82]. RTGN1^T^ also putatively contained the *vanW* and *vanT* genes; however, as these two genes were missing their respective gene clusters to conduct their function, it is not thought these two genes contribute to glycopeptide resistance [83,84].

Resistance to glycopeptide antibiotics in RTGN1^T^ would not be unexpected as *Amycolatopsis* are renowned producers of glycopeptide antibiotics such as vancomycin [14]. Additionally, RTGN1^T^ contains a biosynthetic cluster putatively encoding the glycopeptide mannopeptimycin (Table 3), and it would be expected that RTGN1^T^ would display resistance to glycopeptide antibiotics to allow the production of them [85]. This resistance was confirmed *in vitro* based on the Biolog data as RTGN1^T^ displayed resistance to vancomycin (Table S4).

**Table 3.** The complete list of biosynthetic clusters identified by antiSMASH in *Amycolatopsis* sp. RTGN1^T^

Cluster type	**% identity for each cluster**
Amycolamycin A/amycolamycin B	48
Arsono-polyketide	12
Atratumycin	26
Brasilinolide A/brasilinolide B/brasilinolide C	8
Chlorothricin/deschlorothricin	9
Dutomycin	8
Ectoine	100
Ery-9/Ery-6/Ery-8/Ery-7/Ery-5/Ery-4/Ery-3	100
Fortimicin	4
Friulimicin A/friulimicin B/ friulimicin C/ friulimicin D	21
Funisamine	12
Geosmin	100
Herboxidiene	2
Isorenieratene	25
Kanamycin	2
Lankacidin C	13, 26
Linfuranone B/linfuranone C	38
Macrotermycins	69
Mannopeptimycin	29
Niphimycins C–E	12
Rifamorpholine A/rifamorpholine B/rifamorpholine C/rifamorpholine D/rifamorpholine E	9
Rimosamide	21
Scabichelin	80
Thiolutin	12
TLN-05220	36
Vicenistatin	20
2-Methylisoborneol	50
**No known clusters matching**	**Number of clusters**
Lanthipeptide-class-II	1
Redox cofactor	1
RiPP like	2
NRPS like, other	1
NAPAA	1
Lanthipeptide-class-I	1
RRE containing	2
hglE-KS, type I PKS	1
Total cluster numbers	38

RTGN1^T^ seemed to contain a *Streptomyces rimosus otr(A*) gene, with this gene conferring resistance to tetracycline and possibly aminoglycosides by acting either as an elongation factor to improve translations or a ribosome protectant [86,87]. Based on the Biolog data, RTGN1^T^ was able to grow in the presence of the tetracycline antibiotic, minocycline (Table S4). Resistance to such antibiotics would not be unexpected in RTGN1^T^ as *Amycolatopsis* such as *Amycolatopsis sulphurea* produce tetracycline-like compounds such as cetocycline [88].

### Biosynthetic potential analysis

As annotated by the antiSMASH pipeline, RTGN1^T^ contains 38 clusters putatively encoding secondary metabolites (Table S5), with ten of these clusters not matching to any identified within the database (Table 3). Regarding the other clusters, three had 100% identity, with these putatively encoding ectoine, Ery-9/Ery-6/Ery-8/Ery-7/Ery-5/Ery-4/Ery-3 and geosmin. The other 25 clusters had percentage identity ranging from 2 to 80 %. The ten clusters showing no known matches and the average percentage identity value of the 25 clusters being relatively low indicate the biotechnological potential of RTGN1^T^. This is particularly important regarding the development of novel antimicrobials to combat antibiotic resistance, with RTGN1^T^ containing 16 biosynthetic clusters putatively encoding clusters resembling those that encode the antimicrobials: macrotermycins, TLN-05220, lankacidin C, mannopeptimycin, friulimicins A–D, atratumycin, niphimycins C–E, thiolutin, funisamine, kanamycin, rifamorpholines A–E, dutomycin, brasilinolides A–C, fortimicin, vicenistatin and chlorothricin/deschlorothricin (Table 3). However, due to the majority of these clusters having low identity to the reference clusters, it is possible these clusters encode variants or novel antimicrobials, which may potentially have novel modes of action.

The culture supernatant of RTGN1^T^ was screened using LC-MS to locate useful metabolites. Of those compounds highly expressed, it was noted that RTGN1^T^ seemed to produce several compounds, which may be useful in antimicrobial and biocontrol applications (Table 4). Lauryldimethylamine oxide was produced by RTGN1^T^, with this compound noted as displaying surfactant and antimicrobial activity against Gram-negative and Gram-positive bacteria, akin to other C_12_ amine oxides [89]. Likewise, RTGN1^T^ seemed to produce 3-phenyllactic acid, dl-p-hydroxyphenyllactic acid and 2-hydroxyisocaproic acid, which exhibit antimicrobial activity [90,92].

**Table 4.** Compounds of interest highly expressed (>50%) in the culture supernatant of *Amycolatopsis* sp. RTGN1^T^ relative to the uninoculated media control. Compounds are grouped based upon which function they are associated with

Phosphate solubilization	Percentage (%)
d-(+)-Malic acid	54.74
Gluconic acid	764.25
d-gluconic acid	373.90
Malonic acid	87.71
**Indole compounds**	
Indole-3-carboxylic acid	361.88
**Antimicrobial and biocontrol compounds**	
Lauryldimethylamine oxide	73.49
4-Hydroxyquinoline	352.65
3-Phenyllactic acid	171.04
dl-p-Hydroxyphenyllactic acid	121.81
2-Hydroxyisocaproic acid	182.37
**Miscellaneous PGP-related compounds**	
Putrescine	1,252.89
Isopentenyladenine	63.30
**Other useful secondary metabolites**	
Δ-Aminolevulinic acid	96.19
Levulinic acid	251.55
Paeonol	425.13
p-Coumaric acid	216.62

In addition to antimicrobials directly, RTGN1^T^ produced 4-hydroxyquinoline, a tautomer of 4-quinolone. This compound is noted as a core precursor for many quinolone antibiotics, which may be too large to be captured in LC-MS analysis [93]. Based on the antiSMASH analysis, RTGN1^T^ putatively produces dutomycin, which contains a 4-hydroxyquinoline group (Table 4); thus, produced 4-hydroxyquinoline could serve in the synthesis of this compound [94].

Aside from antimicrobials, based on the LC-MS analysis, RTGN1^T^ was noted as producing Δ-aminolevulinic acid, which displays herbicidal and insecticidal activity [95]. Related to this, levulinic acid, a precursor to Δ-aminolevulinic acid, is produced by RTGN1^T^, which serves as the precursor of a range of other industrial useful compounds [96] (Table 4). Additionally, RTGN1^T^ seemed to produce paeonol, which is noted as having potential benefits in Alzheimer’s disease treatment, preventative treatment for cerebral infarction and putative antimutagenic properties [97,98]. RTGN1^T^ also exhibited production of p-coumaric acid, which, alongside its derivatives and conjugates, are noted as displaying a range of activities related to diabetes management, anti-inflammatory activity, anti-cancer activity, antimicrobial activity and antioxidant activity [99].

### PGP ability assessment

RTGN1^T^ did not appear to possess any genes related to nitrogen fixation based on the IMG and RAST annotation. This would not be unexpected as no other *Amycolatopsis* species fix atmospheric nitrogen. However, RTGN1^T^ did appear to potentially solubilize phosphate based on the presence of *phoABHUR* genes, with one copy of the *phoAR* genes, two copies of the *phoH* gene and three copies of the *phoBU* genes present [100,101]. Based on the literature, it is unknown if other *Amycolatopsis* species contain the pho regulon, as such comparisons in this regard cannot be made.

When phosphate solubilization was investigated *in vitro* using NBRIP media, no large clear zones around RTGN1^T^ were observed, which would be indicative of phosphate solubilization activity. Only small clear zones (0.5–1 mm from the colony edge) were present. Other *Amycolatopsis* such as *Amycolatopsis* sp. 1119 exhibit phosphate solubilization [102], matching the small amount of *in vitro* activity exhibited by RTGN1^T^ upon the NBRIP media. The lack of phosphate solubilization activity may be due to the source of phosphate that was utilized in the NBRIP media. As it has been noted, in other organisms, differing sources of phosphate can elicit differing degrees of phosphate solubilization activity [103]. In addition, it was observed that RTGN1^T^ produces organic acids such as d-(+)-malic acid, gluconic acid, d-gluconic acid and malonic acid (Table 4), which may be utilized in phosphate solubilization as reported in previous investigations [104,106].

According to antiSMASH, RTGN1^T^ may produce the siderophore scabichelin (80% identity), which is also produced by other *Amycolatopsis* such as *Amycolatopsis solani* MEP2-6^T^ [107]. The ability to produce siderophores was expected as many other members of the *Amycolatopsis* genus produce siderophores such as albachelin and albisporachelin [14]. The identity of the putative scabichelin cluster within RTGN1^T^ was relatively high to the scabichelin reference cluster (80%), which was the same identity of the scabichelin cluster found in *A. solani* MEP2-6^T^ [107]. The ability of RTGN1^T^ to produce siderophores *in vitro* was confirmed via a CAS assay, which displayed a large halo of activity around RTGN1^T^ ranging between 42 and 65 mm in diameter after 2 weeks of incubation.

Regarding phytohormones, the RTGN1^T^ genome assembly did not contain the complete gene repertoire for any of the four common pathways, which facilitate the synthesis of IAA, as annotated by IMG [108]. This suggests that RTGN1^T^ is unable to produce IAA, which was confirmed *in vitro*, with no IAA production detected from tryptophan metabolism. This was not unexpected as species within the *Amycolatopsis* genus have been noted as not displaying the ability to produce IAA [109,111]. In a similar manner to IAA, it appeared that RTGN1^T^ did not contain the genes required for the production of gibberellins. This was not unexpected as this does not appear to be a common feature of the genus, with only *Amycolatopsis* sp. SND-1 being reported to produce gibberellins [110].

Based on LC-MS analysis, RTGN1^T^ was noted as producing indole-3-carboxylic acid (Table 4). This compound was found to control phytopathogens such as tobacco mosaic virus and upregulate plant defence signalling, rather than directly promote plant growth [112]. However, RTGN1^T^ may be able to increase the production of indole compounds and gibberellins in the host plant, through the production of putrescine (Table 4). This is based on the exogenous application of putrescine increasing plant growth through the upregulated synthesis of these compounds [113].

Also found in the LC-MS analysis was the cytokinin isopentenyladenine (Table 4). This compound has demonstrated the ability to reduce chlorophyll degradation, promote shoot regeneration, reduce transcription factors related to senescence and upregulate genes related to chloroplasts and ribosomal subunit synthesis [114]. It is unknown if other *Amycolatopsis* produce isopentenyladenine as there is no literature detailing this. However, *Amycolatopsis* isolates such as *Amycolatopsis* sp. SND-1 are noted producers of other cytokinins [110].

Finally, regarding phytohormones, RTGN1^T^ may be able to degrade the precursor of ethylene, ACC, as excess ethylene displays a deleterious effect on plant growth. A gene encoding the enzyme responsible for such degradation, ACC deaminase, was located in the genome of RTGN1^T^ by blasting an ACC deaminase gene sequence from *A. camponoti* (GenBank ID: VVJ21220.1) against the genome of RTGN1^T^, returning a gene annotated as ACC deaminase, with 92% identity. The ability to utilize ACC as a sole carbon source was confirmed *in vitro*, with RTGN1^T^ showing moderate growth upon the media and resulting in relatively strong colour change in the pH indicators.

In addition to ACC deaminase, putrescine produced by RTGN1^T^ may lower host plant ethylene levels. As putrescine has been utilized to extend the shelf life of fruit due to its ability to delay ripening, through lowered ethylene levels, putrescine inhibits the ACC synthase enzyme [115].

## Conclusion

Our polyphasic study revealed that strain RTGN1^T^ represents a novel species within the genus *Amycolatopsis*, isolated from root nodules of *A. glutinosa*. This discovery adds to the limited number of non-*Frankiaceae* species reported from nodules of actinorhizal plants. Through a combination of *in vitro* and *in silico* analyses, we demonstrated that nodule-derived isolates like RTGN1^T^ exhibit significant plant growth-promoting potential, which could be leveraged to develop bioinoculants aimed at enhancing the growth and health of their host actinorhizal plants. Such applications hold promise for improving bioremediation processes by increasing the ecological functionality of these plants.

Moreover, RTGN1^T^ exhibited remarkable biotechnological potential, characterized by a diverse repertoire of putative secondary metabolite biosynthetic gene clusters. Notably, many of these clusters showed low similarity to entries in reference databases, suggesting the presence of novel bioactive compounds. These findings highlight the importance of further exploring this niche for the discovery of new pharmaceutical agents and economically valuable secondary metabolites.

## Description of *Amycolatopsis ponsaeliensis* sp. nov.

*Amycolatopsis ponsaeliensis* (pons.ae.li.en’sis. N.L. fem. adj. *ponsaeliensis*, pertaining to Pons Aelius, the Roman name of Newcastle upon Tyne, where the species was characterized).

Upon GYM *Streptomyces* agar, the colony surface is a dark yellow–brown developing into a white colour as sporulation occurs, and the colony rear is a yellow–gold colour. The colonies have a dry texture with a powdery appearance upon sporulation, and the colonies are small, with round or somewhat irregular form, with a flat elevation and white hyphal margins. Growth was observed between the temperature and pH ranges of 12–28 °C and pH 6–8, respectively, with the optimum being 28 °C and pH 7. According to the Biolog system, it tolerates up to 4% NaCl (w/v), but no growth was observed at 8% NaCl (w/v). Based on *in silico* chemotaxonomy, it may produce phosphatidylethanolamine and phosphatidylglycerol.

According to the Biolog system, it utilizes dextrin, d-maltose, d-trehalose, d-cellobiose, *β-*gentiobiose, sucrose, turanose, d-raffinose, *β*-methyl-d-glucoside, d-salicin, d-glucose, d-mannose, d-fructose, d-galactose, l-rhamnose, d-serine, d-sorbitol, D-arabitol, myo-inositol, pectin, d-galacturonic acid, l-galactonic acid-*γ*-lactone, d-glucuronic acid, d-saccharic acid, citric acid, l-malic acid, bromo-succinic acid, Tween 40 and acetic acid, but not stachyose, *α*-d-lactose, d-melibiose, *N*-acetyl-d-glucosamine, *N*-acetyl-*β*-d-mannosamine, *N*-acetyl-d-galactosamine, *N*-acetyl-neuraminic acid, 3-*O*-methyl-_D_-glucose, d-fucose, l-fucose, inosine, d-mannitol, glycerol, d-glucose-6-phosphate, d-fructose-6-phosphate, d-aspartic acid, gelatin, glycyl-l-proline, l-alanine, l-arginine, l-aspartic acid, l-histidine, l-pyroglutamic acid, l-serine, mucic acid, quinic acid, *p*-hydroxy-phenylacetic acid, methyl pyruvate, d-lactic acid methyl ester, l-lactic acid, d-malic acid, *α*-hydroxy-butyric acid, *β*-hydroxy-butyric acid, *α*-keto-butyric acid, acetoacetic acid and sodium formate.

The genome of RTGN1^T^ has a G+C content of 70.9% and was 11,990,019 bp in length. It was predicted to contain 11,499 genes in total, of which 11,345 were protein coding with 8,221 of these producing a protein with a functional prediction. The draft genome, 16S rRNA gene sequence and SRA data of RTGN1^T^ have been deposited in INSDC under the accession numbers JAPZLD000000000, OP727617 and SRR22101359, respectively. The IMG accession number for the fully annotated draft genome is 2974268288. This species was deposited within the Spanish Type Culture Collection (CECT) and the Centre for Agricultural and Bioscience International (CABI) under the accession numbers CECT 30870 and 507287, respectively.

## Supplementary material

10.1099/ijsem.0.006810 Uncited Supplementary Material 1.

## Abbreviations

ACC: 1-aminocyclopropane-1-carboxylic acid
ANI: average nucleotide identity
blast: Basic Local Alignment Search Tool
CARD: Comprehensive Antibiotic Resistance Database
CAS: chrome azurol S
CRISPR: clustered regularly interspaced short palindromic repeats
dDDH: digital DNA–DNA hybridization
IAA: indole-3-acetic acid
KEGG: Kyoto Encyclopedia of Genes and Genomes
LC-MS: liquid chromatography MS
muscle: Multiple Sequence Comparison by Log-Expectation
NBRIP: National Botanical Research Institute’s phosphate growth
PGP: plant growth promotion
RAST: Rapid Annotation using Subsystem Technology
RGI: Resistance Gene Identifier
TYGS: Type Strain Genome Server
UniProt: Universal Protein
