# Supplementary material for: Amycolatopsis ponsaeliensis sp. nov., a novel endophytic actinobacterium isolated from the root nodules of Alnus glutinosa
Source: Int J Syst Evol Microbiol. 2025 Jun 13;75(6):006810. doi: 10.1099/ijsem.0.006810 (PMC12282000; doi:10.1099/ijsem.0.006810)
Supplement: Uncited Supplementary Material 1. [file ijsem-75-06810-s001.pdf]

Amycolatopsis ponsaeliensis* sp. nov., a novel endophytic actinobacterium isolated from the root nodules of *Alnus glutinosa

Ryan Michael Thompson[a]*, Edward M. Fox[b], Georgios Koutsidis[b], Maria del Carmen Montero-Calasanz[a,c]*

[a]School of Natural and Environmental Sciences, Newcastle University, Newcastle upon Tyne, United Kingdom, NE1 7RU.

[b]Department of Applied Sciences, Northumbria University, Newcastle upon Tyne, United Kingdom, NE1 8ST. [c]IFAPA Las Torres-Andalusian Institute of Agricultural and Fisheries Research and Training, Junta de Andalucía, Cra. Sevilla-Cazalla, km 12.2. 41200, Alcalá del Río, Seville, Spain.

*Corresponding authors. E-mail address R.thompson12@newcastle.ac.uk (R.M. Thompson); mariac.montero.calasanz@juntadeandalucia.es (Maria del Carmen Montero-Calasanz).

Supplementary Table S1. 16S rRNA gene similarity values for the 25 validly named species that are closest in relation to RTGN1^T.

Species name	Percentage 16S rRNA similarity value to RTGN1^T
Amycolatopsis rhabdoformis SB026^T	98.96
Amycolatopsis camponotiI A23^T	98.94
Amycolatopsis solani MEP2-6^T	98.87
Amycolatopsis pretoriensis DSM 44654^T	98.51
Amycolatopsis kentuckyensis NRRL B-24129^T	98.51
Amycolatopsis rifamycinica DSM 46095^T	98.51
Amycolatopsis bullii SF27^T	98.51
Amycolatopsis lexingtonensis NRRL B-24131^T	98.37
Amycolatopsis eburnea GLM-1^T	98.37
Amycolatopsis tolypomycina DSM 44544^T	98.30
Amycolatopsis mongoliensis 4-36^T	98.23
Amycolatopsis vancoresmycina DSM 44592^T	98.02
Amycolatopsis vastitatis H5^T	97.92
Amycolatopsis silviterrae C12CA1^T	97.88
Amycolatopsis plumensis SBHS Strp1^T	97.88
Amycolatopsis balhimycina FH 1894^T	97.81
Amycolatopsis iheyensis OK19-0408^T	97.77
Amycolatopsis mediterranei IMSNU 20056^T	97.73
Amycolatopsis stemonae ST1-08^T	97.60
Amycolatopsis dendrobii DR6-1^T	97.46
Amycolatopsis lurida DSM 43134^T	97.43
Amycolatopsis equina SE(8)3^T	97.42
Amycolatopsis hippodrome S3-6^T	97.39
Amycolatopsis rubida DSM 44637^T	97.38
Amycolatopsis australiensis DSM 44671^T	97.31

Supplementary Table S2. Protein sequences used to locate genes within the draft genome of RTGN1^T. Also noted is the gene which is being search for the in putative novel species using the query sequences derived from GenBank, with the percentage identity and annotation of the resulting sequences in the putative novel species presented.

Gene product searched for in the putative novel species	Query sequences	Annotation of the query sequence used in BLAST	GenBank reference	Percentage identity
		***Amycolatopsis* sp. RTGN1^T**		
menJ	*Amycolatopsis* sp. CA-230715	Menaquinone reductase	QWF79846.1	86%
fabI	*Amycolatopsis xylanica* CPCC 202699	Enoyl-[acyl-carrier-protein] reductase [NADH]	SDW53129.1	86%
Fatty acyl-ACP thioesterase B	*Actinosynnema mirum* DSM 43827	Oleoyl-(acyl-carrier-protein) hydrolase	ACU37867.1	74%
1-acyl-sn-glycerol-3-phosphate acyltransferase	*Amycolatopsis orientalis* CPCC 200066	Acyl-phosphate glycerol-3-phosphate acyltransferase	ANN16347.1	85%
Phosphatidyl-glycerophosphatase B (EC: 3.1.3.27)	*Amycolatopsis* sp. M39	Phosphatidylglycerophosphatase B	A4R44_01820	77%
glycosyltransferase	*Amycolatopsis camponoti* A23	peptidoglycan glycosyltransferase	VVJ22938.1	94%

Supplementary Table S3. CRISPR related genes located in the genome of RTGN1^T using CRISPRFinder.

Contig number	CRISPR start-end position	DR length	Spacer number	Designation by CRISPRFinder
1	264695 - 264790	25	1	Possible
3	159859 - 159930	23	1	Possible
3	160084 - 160226	23	2	Possible
5	43857 - 43946	26	1	Possible
5	44064 - 44149	26	1	Possible
7	81216 - 82093	28	14	Confirmed
7	83642 - 83912	28	4	Confirmed
7	91730 - 92243	24	8	Confirmed
7	99597 - 99986	24	6	Confirmed
7	103969 - 104975	24	16	Confirmed
7	109149 - 109420	28	4	Confirmed
7	108907 - 109057	28	2	Possible
8	14386 - 14474	24	1	Possible
11	188208 - 188294	24	1	Possible
11	217573 - 217699	34	1	Possible
11	223082 - 223233	23	2	Possible
12	22722 - 23123	23	6	Confirmed
12	34625 - 34701	26	1	Possible
20	34409 - 34508	37	1	Possible
26	33366 - 33581	24	3	Confirmed
31	83807 - 83941	46	1	Possible
46	80463 - 80550	31	1	Possible
54	66030 - 67006	26	7	Possible
62	21455 - 21549	23	1	Possible
64	50627 - 50805	24	2	Possible

Supplementary Table S4. Carbon and nitrogen source utilisation of *Amycolatopsis* sp. RTGN1^{T}, alongside ability to tolerate the presence of abiotic stressors such as inhibitory compounds, salinity, and antibiotics. +, positive reaction, -, negative reaction, +/-, ambiguous reaction.

Compound	Response	Compound	Response	Compound	Response	Compound	Response
Negative Control	-	D-Raffinose	+	D-Glucose	+	D-Sorbitol	+
Dextrin	+	α-D-Lactose	-	D-Mannose	+	D-Mannitol	-
D-Maltose	+	D-Melibiose	-	D-Fructose	+	D-Arabitol	+
D-Trehalose	+	β-Methyl-D-Glucoside	+	D-Galactose	+	myo-Inositol	+
D-Cellobiose	+	D-Salicin	+	3-O-Methyl-D-Glucose	-	Glycerol	-
β-Gentiobiose	+	N-Acetyl-D-Glucosamine	-	D-Fucose	-	D-Glucose-6-Phosphate	-
Sucrose	+	N-Acetyl-β-D-Mannosamine	-	L-Fucose	-	D-Fructose-6-Phosphate	-
Turanose	+	N-Acetyl-D-Galactosamine	-	L-Rhamnose	+	D-Aspartic Acid	-
Stachyose	-	N-Acetyl-Neuraminic Acid	-	Inosine	-	D-Serine #1	-
Positive Control	+	1% NaCl)	+	1% Sodium Lactate	-	Troleandomycin	+
pH 6	+/-	4% NaCl	+	Fusidic Acid	+	Rifamycin SV	+
pH 5	+	8% NaCl	+/-	D-Serine #2	+	Minocycline	+

Compound	Response	Compound	Response	Compound	Response	Compound	Response
Gelatin	-	Pectin	+	p-Hydroxy-Phenylacetic Acid	-	Tween 40	+
Gly-Pro	-	D-Galacturonic Acid	+	Methyl Pyruvate	-	γ-Amino-n-Butyric Acid	+/-
L-Alanine	-	L-Galactonic Acid-γ-Lactone	+	D-Lactic Acid Methyl Ester	-	α-Hydroxy-Butyric Acid	-
L-Arginine	-	D-Gluconic Acid	+/-	L-Lactic Acid	-	β-Hydroxy-Butyric Acid	-
L-Aspartic Acid	-	D-Glucuronic Acid	+	Citric Acid	+	α-Keto-Butyric Acid	-
L-Glutamic Acid	+/-	Glucuronamide	+/-	α-Keto-Glutaric Acid	+/-	Acetoacetic Acid	-
L-Histidine	-	Mucic Acid	-	D-Malic Acid	-	Propionic Acid	+/-
L-Pyroglutamic Acid	-	Quinic Acid	-	L-Malic Acid	+	Acetic Acid	+
L-Serine	-	D-Saccharic Acid	+	Bromo-Succinic Acid	+	Sodium Formate	-

Lincomycin	+	Vancomycin	+	Nalidixic Acid	+	Aztreonam	+
Guanidine Hydrochloride	-	Tetrazolium Violet	-	Lithium Chloride	-	Butyric Acid	+
Niaproof	-	Tetrazolium Blue	-	Potassium Tellurite	+	Sodium Bromate	+

Supplementary Table S5. Biosynthetic gene clusters detected using antiSMASH.

Biosynthetic cluster type	Most similar known cluster	Percent similarity
redox-cofactor	lankacidin C	13 %
lanthipeptide-class-II	-	-
PKS-like	arsono-polyketide	12 %
PKS-like	herboxidiene	2 %
NRPS	mannopeptimycin	29 %
redox-cofactor	-	-
NRPS, lanthipeptide-class-II	friulimicin A/friulimicin B/ friulimicin C/ friulimicin D	21 %
Ladderane, NRPS, NRPS-like	atratumycin	26 %
Terpene	isorenieratene	25 %
Terpene	2-methylisoborneol	50 %
RiPP-like	-	-
Type I PKS, NRPS-like	niphimycins C-E	12 %
Type II PKS, other, aminocoumarin	TLN-05220	36 %
NRPS-like, other	-	-
hglE-KS	thiolutin	12 %
NAPAA	-	-
NRPS-like	funisamine	12 %
redox-cofactor	lankacidin C	26 %
lanthipeptide-class-III	Ery-9/Ery-6/Ery-8/Ery-7/Ery-5/Ery-4/Ery-3	100 %
Indole	kanamycin	2 %
RiPP-like	-	-
transAT-PKS, hglE-KS, Type I PKS	rifamorpholine A/rifamorpholineB/rifamorpholineC/rifamorpholine D/rifamorpholine E	9 %
Type II PKS	dutomycin	8 %
NRPS, nucleoside	rimosamide	21 %
NRPS	scabichelin	80 %
Type I PKS, butyrolactone	macrotermycins	69 %
Ectoine	ectoine	100 %
Terpene	geosmin	100 %
lanthipeptide-class-I	-	-
Type I PKS,PKS-like, oligosaccharide	brasilinolide A/brasilinolide B/brasilinolide C	8 %
Indole	fortimicin	4 %
RRE-containing, Type I PKS,NRPS,NRPS-like	amycolamycin A/amycolamycin B	48 %
RRE-containing	-	-
hglE-KS, Type I PKS	-	-
Type I PKS	linfuranone B/linfuranone C	38 %
Type I PKS	vicenistatin	20 %
Aminocoumarin, Type I PKS	chlorothricin/deschlorothricin	9 %
RRE-containing	-	-
